# Deep Learning-Based Min-Entropy-Accelerated Evaluation for High-Speed Quantum Random Number Generation

**DOI:** 10.3390/e27080786

**Published:** 2025-07-24

**Authors:** Xiaomin Guo, Wenhe Zhou, Yue Luo, Xiangyu Meng, Jiamin Li, Yaoxing Bian, Yanqiang Guo, Liantuan Xiao

**Affiliations:** 1 Key Laboratory of Advanced Transducers and Intelligent Control System, Ministry of Education, Taiyuan University of Technology, Taiyuan 030024, China; guoxiaomin@tyut.edu.cn (X.G.); zhouwenhe1171@link.tyut.edu.cn (W.Z.); luoyue1508@link.tyut.edu.cn (Y.L.); mengxiangyu1511@link.tyut.edu.cn (X.M.); 2 College of Physics and Optoelectronics, Taiyuan University of Technology, Taiyuan 030024, China; lijiamin@tyut.edu.cn (J.L.); bianyaoxing@tyut.edu.cn (Y.B.); 3 Shanxi Key Laboratory of Precision Measurement Physics, Taiyuan University of Technology, Taiyuan 030024, China

**Keywords:** quantum random number generation, quantum conditional min-entropy, dual-quadrature heterodyne detection, quantum cryptography, deep convolutional neural network

## Abstract

Secure communication is critically dependent on high-speed and high-security quantum random number generation (QRNG). In this work, we present a responsive approach to enhance the efficiency and security of QRNG by leveraging polarization-controlled heterodyne detection to simultaneously measure the quadrature amplitude and phase fluctuations of vacuum shot noise. To address the practical non-idealities inherent in QRNG systems, we investigate the critical impacts of imbalanced heterodyne detection, amplitude–phase overlap, finite-size effects, and security parameters on quantum conditional min-entropy derived from the entropy uncertainty principle. It effectively mitigates the overestimation of randomness and fortifies the system against potential eavesdropping attacks. For a high-security parameter of $10^{-20}$, QRNG achieves a true random bit extraction ratio of 83.16% with a corresponding real-time speed of 37.25 Gbps following a 16-bit analog-to-digital converter quantization and 1.4 GHz bandwidth extraction. Furthermore, we develop a deep convolutional neural network for rapid and accurate entropy evaluation. The entropy evaluation of 13,473 sets of quadrature data is processed in 68.89 s with a mean absolute percentage error of 0.004, achieving an acceleration of two orders of magnitude in evaluation speed. Extracting the shot noise with full detection bandwidth, the generation rate of QRNG using dual-quadrature heterodyne detection exceeds 85 Gbps. The research contributes to advancing the practical deployment of QRNG and expediting rapid entropy assessment.

## 1. Introduction

Random numbers are indispensable in a multitude of information fields, including cryptographic algorithms, secure communication, Monte Carlo simulations, and financial security [1]. With the rapid development of quantum technology, especially the emergence and application of quantum computers, the encryption security of algorithm-based pseudo-random number generators is confronted with a significant challenge [2,3,4]. Quantum random number generation (QRNG) leverages the inherent unpredictability of quantum mechanics, as dictated by the uncertainty principle, to provide a natural platform for secure random number generation. To date, a variety of quantum random number protocols have been proposed, which can be broadly categorized based on the type of exploited quantum noise: discrete-variable and continuous-variable signals. The former protocols encode information in the physical attributes of single photons, where quantum randomness arises from spatial [5,6], temporal [7,8,9], and photon number distribution [10,11,12,13] properties. The latter protocols encode information in the quadrature observables of continuous-variable light fields, with quantum randomness stemming from vacuum fluctuations [14,15,16,17], phase noise [18,19,20], and amplified spontaneous emission [21,22,23]. Continuous-variable QRNG utilizes wideband photodetectors instead of single-photon detectors, achieving high-speed random bit generation rates up to Gbps. QRNG based on vacuum fluctuations is poised for promising practical development due to its robustness, fast performance, high security, and compatibility with integration. While vacuum-based QRNG is ideally capable of generating unpredictable random numbers, its security is not guaranteed due to potential classical noise or side information that could be exploited by an eavesdropper [24,25,26]. Addressing the practical concerns in one-quadrature-fluctuation extraction systems, quantum conditional min-entropy evaluations [27,28] and semi-device-independent protocols [29,30,31] have been refined to account for component device influences. The advancements in measurement-device-independent [32] and device-independent schemes [33] have notably enhanced the security and robustness of QRNG by mitigating potential vulnerabilities associated with imperfect devices. A heterodyne detection scheme [34,35,36] can offer an approach with faster bit rates and phase space sampling to counteract quantum attacks. In heterodyne detection schemes, a positive operator value measurement model and mutual testing have been proposed to extract secure bits without relying on trusted entropy sources. However, in high-speed QRNG systems, crucial impact factors, including dual-quadrature beam splitting ratio error, local oscillator fluctuations, finite-size effects, and security parameters, limit the whole system’s security and remain to be explored.

Moreover, the existing entropy estimations in QRNG security evaluation are mainly categorized into theoretical modeling, statistical testing, machine learning, and deep learning methods. For the theoretical modeling method, the complexity in principle and structure of many entropy sources often precludes accurate modeling [37]. The statistical testing method, involving post-processing examination using statistical test suites after QRNG, is limited by time-consuming computations and unsuitable for fast entropy source assessment [38]. Typical deep-learning evaluation methods assess QRNG security by comparing benchmark probabilities with predicted probabilities. It transforms the evaluation issue of QRNG randomness or unpredictability into a problem of neural network model learning, specifically whether the model can learn the underlying correlations or patterns among data [39]. However, entropy quantification for the unpredictability of random numbers, especially the establishment of a more rigorous practical entropy evaluation model, remains to be further developed, and performing efficient online entropy assessment across multiple datasets and devices continues to be a key challenge.

In this work, we experimentally present a high-speed online QRNG and entropy evaluation based on deep learning-based heterodyne detection of vacuum shot noise in quadrature amplitude and phase fluctuations. To enhance the robustness of the QRNG system, we employ polarizing beam splitters and controllers. The imperfect impacts of imbalanced heterodyne detection, amplitude–phase overlap, finite-size effects, and security parameters are investigated. Furthermore, we develop a practical security evaluation protocol by ascertaining a rigorous lower bound of quantum conditional min-entropy, enabling the system to autonomously mitigate source and measurement defects. Following 16-bit analog-to-digital converter (ADC) quantization and broadband shot-noise extraction above the GHz range, we achieve an ultrafast QRNG with a high-security parameter and a high random bit extraction ratio. For rapid and accurate entropy evaluation, a deep convolutional neural network (DCNN) is developed, achieving precise mapping across a wide range of entropy variations. Compared to other deep learning methods, the developed DCNN achieves a higher accuracy and speed entropy evaluation with a negligible mean absolute percentage error (MAPE). The entropy evaluation speed is enhanced by two orders of magnitude over non-deep learning methods. Our scheme not only provides a promising framework for QRNG operating at hundreds of Gbps but also defends against quantum side-information attacks, holding important applications for the practicality of quantum random number generators.

## 2. Proposed Method

QRNG is typically divided into three components: entropy source preparation, positive operator value measurement, and post-processing. In our scheme, raw randomness is generated from the shot noise of vacuum state, assuming the worst case where the entropy source is completely controlled by an eavesdropper. In the positive operator value measurement section, heterodyne measurement is employed and imperfect impacts on the measurement outcomes are quantified. The resulting signals are then directed to the detectors and corresponding signal processing circuits. In the subsequent post-processing stage, a real-time post-processing and a DCNN are utilized to accelerate the extraction of secure quantum random numbers. Following extraction processing, unreliable or biased non-quantum entropy is ultimately eliminated, culminating in the generation of reliable secure quantum random bits.

### 2.1. Dual-Quadrature Heterodyne Detection

**Figure 1:**
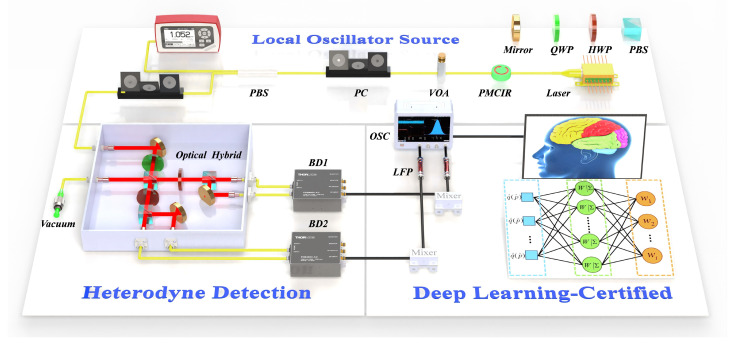
Schematic illustration of experimental setup. Laser: single-mode laser (LO laser); PMCIR: polarization-maintaining fiber optical circulator; PC: polarization controller; VOA: variable optical attenuator; PBS: polarization beam splitter; QWP: quarter-wave plate; HWP: half-wave plate; BD: balanced detection; LPF: low pass filter; OSC: oscilloscope (data acquisition system).

Heterodyne detection, highly sensitive to both the amplitude and phase of incident light, is utilized for measuring the quadrature amplitude and phase fluctuations of quantum shot noise. We also employ an optical hybrid to control the beam polarization and introduce a phase delay, ensuring a 90° phase difference between the outputs in the amplitude Q and phase P quadratures, respectively. This results in a 90° phase difference in the output signals obtained by the balanced detectors. The schematic model is depicted in the “Heterodyne Detection” section of Figure 1, illustrating the precise synthesis of the signal and local oscillator (LO) to produce coherent mixing. The interference between the LO laser and the vacuum signal takes place within the hybrid device. Subsequently, the power spectrum of broadband vacuum shot noise is acquired by the heterodyne detection. To investigate the actual security of heterodyne detection systems, we derive the impact of practical system errors on heterodyne quadrature measurements. The LO and the signal light are denoted by $E_{L}\left(t\right)=E_{L}+\delta Q_{L}\left(t\right)+i\delta P_{L}\left(t\right)$ and $E_{S}\left(t\right)=E_{S}+\delta Q_{S}\left(t\right)+i\delta P_{S}\left(t\right)$, respectively, and $E_{L}\gg E_{S}$. The $E_{L}$ and $E_{S}$ are time-independent, whereas $\delta Q_{L(S)}\left(t\right)$ and $\delta P_{L(S)}\left(t\right)$ denote the time-dependent terms associated with the Q and P quadratures of the LO (signal) field. The light interference occurs in the optical hybrid, and the hybrid outputs from different channels exhibit minor phase discrepancies. Accounting for the influence of non-ideal polarization devices, the imbalance of the polarization element is denoted as l, and the actual phase deviation from the ideal value is represented by π/2+θ. The quantum efficiencies of the photodetectors are denoted by $\eta_{1}$, $\eta_{2}$, $\eta_{3}$, and $\eta_{4}$. We assume that the imbalances of the PBS2 and PBS3 obey $l_{1}=l_{2}=l$, and derive the differential outputs of the two quadratures as follows:

(1) $$\begin{array}{cc} I_{1}\left(t\right) & =\left[\frac{1}{2}\eta_{1}\left(\frac{1}{2}-l\right)-\frac{1}{2}\eta_{2}\left(\frac{1}{2}+l\right)\right]\left(E_{L}^{2}+2E_{L}\delta Q_{L}\left(t\right)\right)\\ \; & +E_{L}\delta Q_{S}\left(t\right)\left(\eta_{1}+\eta_{2}\right)\sqrt{\left(\frac{1}{2}-l\right)\left(\frac{1}{2}+l\right)}, \end{array}$$

(2) $$\begin{array}{cc} I_{2}\left(t\right) & =\left[\frac{1}{2}\eta_{3}\left(\frac{1}{2}-l\right)-\frac{1}{2}\eta_{4}\left(\frac{1}{2}+l\right)\right]\left(E_{L}^{2}+2E_{L}\delta Q_{L}\left(t\right)\right)\\ \; & +E_{L}\left(\delta P_{S}\left(t\right)\cos\theta +\delta Q_{S}\left(t\right)\sin\theta \right)\\ \; & \cdot \left(\eta_{3}+\eta_{4}\right)\sqrt{\left(\frac{1}{2}-l\right)\left(\frac{1}{2}+l\right)}. \end{array}$$

where $I_{1}\left(t\right)$ and $I_{2}\left(t\right)$ represent the quadrature measurement results of Q and P, respectively, $E_{L}^{2}$ represents the bias voltage of the detector, $\delta Q_{L}\left(t\right)$ represents the LO intensity fluctuation, and $\delta P{\left(Q\right)}_{S}\left(t\right)$ represents the quantum fluctuation of the measured signal.

In the derivation process, the higher-order terms of the time-varying related terms $\delta Q_{L(S)}\left(t\right)$ and $\delta P_{L(S)}\left(t\right)$ are all assumed to be 0. From Equations (1) and (2), it can be obtained that the LO fluctuation variance for the Q quadrature is given by

(3) $$\begin{array}{c} \sigma_{LO,Q}^{2}=E_{L}^{2}{\left[\eta_{1}\left(\frac{1}{2}-l\right)-\eta_{2}\left(\frac{1}{2}+l\right)\right]}^{2}D\left[\delta Q_{L}\left(t\right)\right], \end{array}$$

and the quantum noise variance is

(4) $$\begin{array}{c} \sigma_{V,Q}^{2}=E_{L}^{2}{\left(\eta_{1}+\eta_{2}\right)}^{2}\left(\frac{1}{2}-l\right)\left(\frac{1}{2}+l\right)D\left[\delta Q_{S}\left(t\right)\right]. \end{array}$$

For the P quadrature, the LO fluctuation variance is

(5) $$\begin{array}{c} \sigma_{LO,P}^{2}=E_{L}^{2}{\left[\eta_{3}\left(\frac{1}{2}-l\right)-\eta_{4}\left(\frac{1}{2}+l\right)\right]}^{2}D\left[\delta Q_{L}\left(t\right)\right], \end{array}$$

and its quantum noise variance is

(6) $$\begin{array}{cc} \sigma_{V,P}^{2} & =E_{L}^{2}{\left(\eta_{3}+\eta_{4}\right)}^{2}\left(\frac{1}{2}-l\right)\left(\frac{1}{2}+l\right)\\ \; & \cdot (D\left(\delta P_{s}\left(t\right)\right)\cos^{2}\theta +D\left(\delta Q_{s}\left(t\right)\right)\sin^{2}\theta ), \end{array}$$

where $E_{L}^{2}$ equals the LO optical power, denoted as $E_{L}^{2}=P_{LO}$·$D[\delta P_{s}\left(t\right)]$, and $D[\delta Q_{s}\left(t\right)]$ represent the initial variances of vacuum fluctuations, typically denoted as $D\left[\delta P_{s}\left(t\right)\right]=D\left[\delta Q_{s}\left(t\right)\right]=\sigma^{2}$. The $D(\delta Q_{L}\left(t\right))$ denotes the initial variance of the LO fluctuations, denoted as $D\left[\delta Q_{L}\left(t\right)\right]=k\sigma^{2}$. The LO fluctuations can be quantified by the *k*. Considering the variance of electronic noise $\sigma_{E,Q(P)}^{2}$, the measurement value of the signal Q(P) quadrature can be expressed as $\sigma_{M,Q(P)}^{2}=\sigma_{V,Q(P)}^{2}+\sigma_{LO,Q(P)}^{2}+\sigma_{E,Q(P)}^{2}$.

### 2.2. High-Speed QRNG and Entropy Evaluation Setup

Based on the above analysis, we built an experimental setup for the dual-quadrature heterodyne detection, as illustrated in Figure 1. The setup was divided into three main components: polarization-controlled and monitored LO laser, heterodyne measurement of phase and amplitude quadratures, and quantum noise extraction with accelerated entropy evaluation using deep learning techniques. Specifically, a single-mode continuous-wave semiconductor laser with a wavelength of 1550 nm served as the LO source. The LO light passed through a polarization-maintaining fiber optical circulator (PMCIR) with 67 dB high return loss, functioning as a polarization controller and an isolator. The PMCIR effectively suppressed the return of reflected light to the signal port. Then, a variable optical attenuator (VOA) was used to adjust the power of the LO. This ensured the stability of the output laser and determined the optimal power of the LO. A group of a polarization controller (PC) and a fiber polarization beam splitter (PBS) was employed to split the LO light into a 90:10 ratio, with the 10% branch monitored by a power meter for the LO output. The 90% light was injected into a 90° optical hybrid module, where the vacuum state was input through the signal port and the other beam was split and coupled with a 90° phase delay. Prior to entering the optical hybrid, the polarization controllers and VOA were used to precisely adjust and control the amplitude and phase quadratures, ensuring equal input intensities. The outputs were detected by a pair of high-quantum-efficiency and wideband-balanced photodetectors (BD, PDB480C, Thorlabs Inc., Newton, MA, USA) with a 1.6 GHz bandwidth. The two detectors, BD1 and BD2, were of identical specifications and serve different quadrature measurements: BD1 was utilized to measure the amplitude quadrature, while BD2 was employed to measure the phase quadrature. The resulting shot-noise signals with bandwidths of 1.4 GHz were extracted by electrical mixing and filtering. The power spectrum and time sequences of the shot noise were recorded using a 26.5 GHz spectrum analyzer (SA, N9020A, Agilent Technologies Inc., Santa Clara, CA, USA) and a real-time oscilloscope (OSC, LabMaster10-36Zi, Lecroy, NY, USA) with a bandwidth of 36 GHz, respectively. Then, the raw random bits were obtained by 16-bit ADC quantization. Finally, the entropy extraction and evaluation of the random bits were accelerated using a deep learning model.

### 2.3. Security and Quantum Conditional Min-Entropy Evaluation

It is crucial to assess the quantum randomness of the raw data. In order to ensure the security of the system, we estimate and quantify the secure randomness under the condition of an eavesdropper’s attack on the source. Considering the effects of local oscillator fluctuations, imbalanced detection error, phase error, finite-size effects, and security parameters, we investigated the variations in quantum conditional min-entropy over a wide range of non-ideal factors. It can be achieved through the rigorous formalization of the entropy uncertainty relationship [30,40,41]. In the heterodyne detection, quadratures P and Q are measured simultaneously and enable a limit on the extractable randomness. The quantum conditional min-entropy of the heterodyne system based on the entropy uncertainty relationship can be expressed as [36]

(7) $$\begin{array}{cc} H_{\min}\left(M|E\right) & \geq -2\log_{2}c\left(\delta_{q},\delta_{p}\right)-H_{\max}\left(Q_{\delta_{q}}\right)-H_{\max}\left(P_{\delta_{p}}\right), \end{array}$$

(8) $$\begin{array}{c} c\left(\delta_{q},\delta_{p}\right)=\frac{\delta_{q}\delta_{p}}{2\pi }S_{0}^{\left(1\right)}{\left(1,\frac{\delta_{q}\delta_{p}}{4}\right)}^{2}, \end{array}$$

where $c(\delta_{q},\delta_{p})$ represents the overlap degree between the P and Q, $S_{0}^{\left(1\right)}{\left(1,\frac{\delta_{q}\delta_{p}}{4}\right)}^{2}$ is the 0th radial prolate spheroidal wave function of the first kind, $H_{\max}\left(Q_{\delta_{q}}\right)$ and $H_{\max}\left(P_{\delta_{p}}\right)$ denote the maximum entropies of the Q and P, and $H_{\max}\left(Q{\left(P\right)}_{\delta_{q(p)}}\right)=2\log_{2}\sum_{i}\sqrt{p_{dis}\left(q{\left(p\right)}_{i}\right)}$ denotes the max-entropy associated with the quadratures. $\delta_{q}$ and $\delta_{p}$ indicate the measurement accuracies of the P and Q, as given by $\delta =\frac{\delta_{ADC}}{\sqrt{2\sigma_{V}^{2}}}$. $\delta_{ADC}$ signifies the resolution of the ADC.

Due to the non-ideal factors of the splitting ratio of the optical hybrid and the detection efficiency in the realistic experiment, unbalanced detection occurs and misinterprets the fluctuations of the LO light as part of the quantum noise, leading to an erroneous estimation of the measurement resolution. Then, we analyze the above imperfect impact on the measurement accuracy of the Q (P). Assuming the sampling range of the ADC to three standard confidence intervals, namely $N=3\sigma_{M}$, the discrepancy between the actual accuracy and the ideal accuracy is given by

(9) $$\begin{array}{cc} \Delta_{\delta } & =\frac{\delta_{ADC}}{\sqrt{2\sigma_{V}^{2}}}-\frac{\delta_{ADC}}{\sqrt{2(\sigma_{V}^{2}+\sigma_{LO}^{2})}}\\ \; & =\frac{3\sqrt{\left(1+4\left(k-1\right)l^{2}\right)\sigma^{2}P_{LO}+\sigma_{E}^{2}}}{2^{n-1}\sqrt{2P_{LO}\sigma^{2}}}\\ \; & \cdot (\frac{1}{\sqrt{1-4l^{2}}}-\frac{1}{\sqrt{1+4\left(k-1\right)l^{2}}}). \end{array}$$

Therefore, the actual entropy is

(10) $$\begin{array}{cc} H_{\min}^{\delta }\left(M|E\right) & \geq -2\log_{2}c\left(\delta_{q}+\Delta_{\delta },\delta_{p}+\Delta_{\delta }\right)\\ \; & -H_{\max}\left(Q_{\delta_{q}+\Delta_{\delta }}\right)-H_{\max}\left(P_{\delta_{p}+\Delta_{\delta }}\right). \end{array}$$

**Figure 2:**
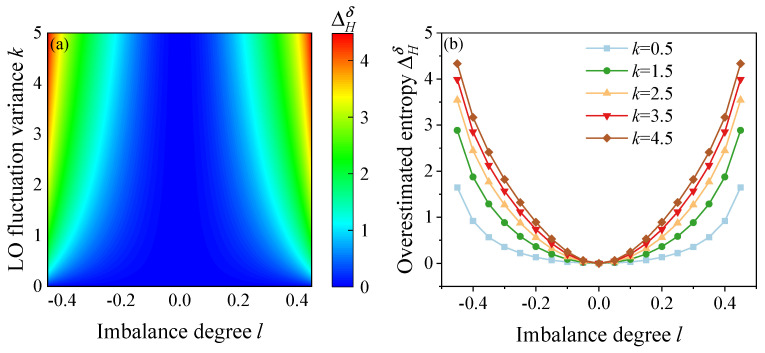
(**a**) Map of $\Delta_{H}^{\delta }$ as a function of *k* and *l*. (**b**) Entropy deviation versus the degree of imbalance *l* when the initial LO variance *k* = 0.5, *k* = 1.5, *k* = 2.5, *k* = 3.5, *k* = 4.5, respectively.

Figure 2a illustrates a map of $\Delta_{H}^{\delta }$ as a function of *k* and *l* when $P_{LO}=1,$n=8,$\sigma_{E,Q(P)}^{2}=0.1,\sigma^{2}=0.5$, where $\Delta_{H}^{\delta }=H_{\min}\left(M|E\right)-H_{\min}^{\delta }\left(M|E\right)$ indicates the overestimated quantum bits under imbalance detection and n is the bit resolution of the ADC. For the *k* ranging from 0 to 5 and the *l* ranging from −0.45 to 0.45, the results reveal that, as the initial LO fluctuation variance *k* and the degree of imbalance *l* increase, the overestimated entropy $\Delta_{H}^{\delta }$ increases, varying from 0 to 4.48 bits. Specifically, Figure 2b depicts the variation in $\Delta_{H}^{\delta }$ with the degree of imbalance *l* for *k* = 0.5, 1.5, 2.5, 3.5, and 4.5.

Furthermore, we investigated the impact of the actual phase deviation θ of the optical hybrid from the ideal value in an unbalanced homodyne detection on the extractable randomness. In practice, the measurements of the quadratures Q and P satisfy the canonical commutation relation $\left[Q,P\right]=i\hbar \cos\theta$ [42], and the actual overlap is given by

(11) $$c^{\prime }\left(\delta_{q},\delta_{p}\right)=c\left(\delta_{q},\delta_{p}\right)/\cos\theta .$$

In this case, the overestimated extractable entropy is

(12) $$\begin{array}{cc} H_{\min}^{\delta ,\theta }\left(M|E\right) & \geq -2\log_{2}\frac{c(\delta_{q}+\Delta_{\delta },\delta_{p}+\Delta_{\delta })}{\cos\theta }\\ \; & -H_{\max}\left(Q_{\delta_{q}+\Delta_{\delta }}\right)-H_{\max}\left(P_{\delta_{p}+\Delta_{\delta }}\right). \end{array}$$

The overestimated quantum random bits are

(13) $$\begin{array}{c} \Delta_{H}^{\delta ,\theta }=H_{\min}\left(M|E\right)-H_{\min}^{\delta ,\theta }\left(M|E\right). \end{array}$$

**Figure 3:**
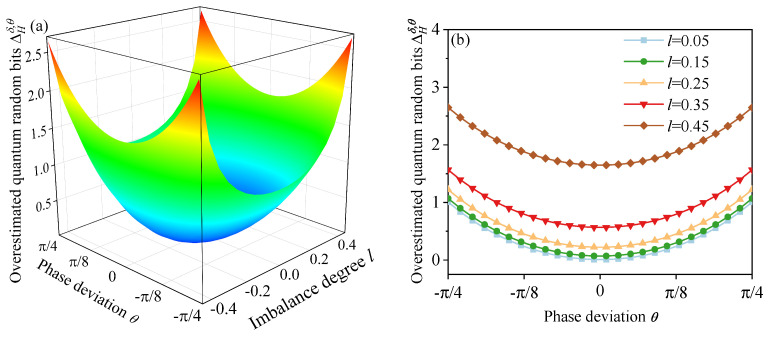
(**a**) Overestimated extractable randomness $\Delta_{H}^{\delta ,\theta }$ versus imbalance degree *l* and phase deviation θ. (**b**) $\Delta_{H}^{\delta ,\theta }$ as a function of θ for imbalance degree l=0.05,0.15,0.25,0.35,0.45, respectively.

Figure 3a illustrates the variation in overestimated quantum random bits $\Delta_{H}^{\delta ,\theta }$ under the effects of imbalance *l* and imperfect phase deviation θ. When the θ gradually changes from −π/4 to π/4, the *l* gradually varies from −0.45 to 0.45. It can be noted that, as the deviation θ and the imbalance *l* increase, the overestimated extractable random bits gradually increase, ranging from 0 to 2.65 bits. Figure 3b shows the variation $\Delta_{H}^{\delta ,\theta }$ with the phase deviation θ when the imbalance degrees l=0.05,0.15,0.25,0.35,0.45, respectively. It is worth noting that the impact of imbalance deviation on the $\Delta_{H}^{\delta ,\theta }$ is higher than that of phase deviation.

**Figure 4:**
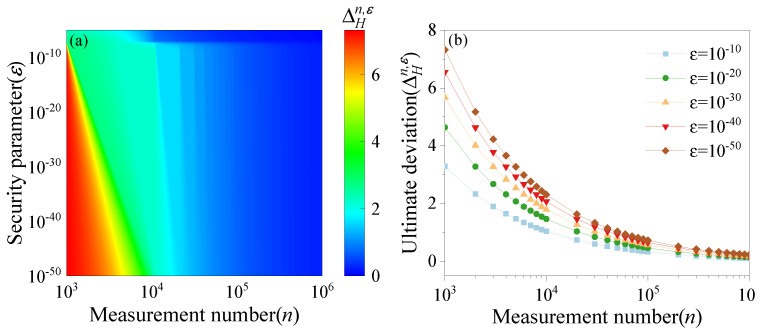
(**a**) Map of $\Delta_{H}^{n,\varepsilon }$ as a function of *n* and ε. (**b**) Entropy deviation versus measurement numbers when the security parameters are $10^{-10},10^{-20},10^{-30},10^{-40},10^{-50}$, respectively.

Finally, considering the above non-ideal factors and the finite-size effects, we establish the bound of quantum conditional min-entropy for source-independent QRNG to be

(14) $$\begin{array}{c} H_{\min}^{\delta ,\theta ,n,\varepsilon }\left(M|E\right)=H_{\min}^{\delta ,\theta }\left(M|E\right)-\Delta_{H}^{n,\varepsilon }, \end{array}$$

(15) $$\begin{array}{cc} \Delta_{H}^{n,\varepsilon } & =\frac{4}{\sqrt{n_{p}}}\sqrt{\log_{2}\left(\frac{2}{\varepsilon^{2}}\right)}\log_{2}\left[2^{1+\frac{H_{\max}\left(Q_{\delta_{q}+\Delta_{\delta }}\right)}{2}}+1\right]\\ \; & +\frac{4}{\sqrt{n_{q}}}\sqrt{\log_{2}\left(\frac{2}{\varepsilon^{2}}\right)}\log_{2}\left[2^{1+\frac{H_{\max}\left(P_{\delta_{p}+\Delta_{\delta }}\right)}{2}}+1\right], \end{array}$$

where *n* denotes the number of measured data points obtained from each quadrature setting, and ε denotes the security parameter. To investigate the two impacts on the overall entropy deviation, Figure 4a shows the map of $\Delta_{H}^{n,\varepsilon }$ as a function of *n* and ε. Figure 4b shows the variation in $\Delta_{H}^{n,\varepsilon }$ with the measurement number *n* when the security parameter $\varepsilon =10^{-10},10^{-20},10^{-30},10^{-40},10^{-50}$, respectively. It can be seen that the overestimated quantum random bits decrease with an increase in the measurement number, and, when the measurement number reaches a $10^{6}$ order of magnitude, the influence of *n* on the ultimate deviation $\Delta_{H}^{n,\varepsilon }$ is insignificant.

### 2.4. Deep Learning Method

With the advancement in computer arithmetic power and the development of artificial intelligence, machine learning and deep learning methods have begun to be used for secure communication. The applications include security analysis of key distribution and random number generators, communication protocol optimization, noise suppression, and error correction [43,44,45]. Among them, convolutional neural networks demonstrate outstanding ability in pattern recognition and feature extraction. In our QRNG, we focus on rapid and precise entropy evaluation when multiple entropy sources and multiple devices operate concurrently. We develop a deep convolutional neural network (DCNN) capable of automatically learning the min-entropy features of the quadrature fluctuations. The DCNN takes the collected quadrature measurements as input $x=(x_{1},x_{2},x_{3}\cdots )$ and after model training outputs $y(H_{\min}\left(X|E\right)|x_{1},x_{2},x_{3}\cdots )$ with the aim of establishing the relationship between $H_{\min}\left(X|E\right)$ and the quadrature fluctuations. The DCNN is trained on the model associated with Equation (14).

To enhance the accuracy and estimation speed of the DCNN, data preprocessing is essential. Initially, the dataset is divided into training and testing sets, with the training set comprising 80% of the data and the testing set accounting for the remaining 20%. The dataset contains a wide range of power variations, with the training and testing data exhibiting substantial diversity in power range and signal variation. Subsequently, all samples are labeled by taking a continuous sequence set of 10,000 vacuum fluctuation data as the input and the corresponding min-entropy value at that stage as the label. The dataset is then sequentially shifted backward by 1000 samples to form the next input sequence. This shifting process is continued until all input sequences and corresponding labels are generated. The final set of inputs and labels is then shuffled to be used for subsequent model training phases. It should be noted that these datasets are collected under different quantum conditional min-entropy scenarios. Our DCNN model structure involves the initial input features passing through a one-dimensional convolutional layer with 32 channels using a convolutional kernel size of 3 with a stride of 1. After passing through a max pooling layer, the features proceed to the subsequent convolutional layers. Specifically, the second convolutional layer comprises 64 channels, the third layer comprises 128 channels, and the fourth layer comprises 64 channels. Each convolutional layer, including the initial one, employs Rectified Linear Unit (ReLU) activation functions. Pooling layers are interspersed between the convolutional layers to perform down-sampling, which aids in extracting the primary information from the input features, suppressing noise and irrelevant details, and mitigating the risk of overfitting. The final output is derived from two fully connected layers, where the first layer accepts 20,000 input features and outputs 1000 features and the second layer receives 1000 input features and produces a single output feature. During model training, the batch size is set to 32, and the model is optimized employing stochastic gradient descent (SGD) with a learning rate of 0.0001. The maximum number of training epochs is set to 200. In the supervised training process, the labels are used to assess the accuracy and other critical metrics of the model. The deep learning is implemented based on Keras and the backend of PyTorch with Python and executed on a Windows system equipped with an Intel i5-10400X CPU and an NVIDIA RTX 2080 Ti GPU.

## 3. Experimental Results

### 3.1. High-Speed and High-Security QRNG Using Dual-Quadrature Heterodyne Detection

**Figure 5:**
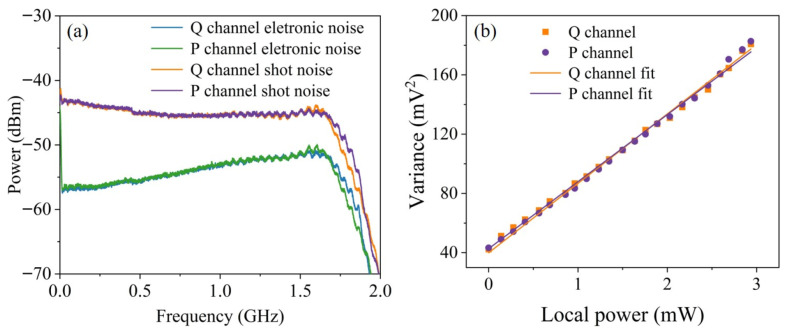
(**a**) Power spectrum of quantum shot noise when the LO power is 3 mW. (**b**) Linear dependence of the signal quadrature $\sigma_{V}^{2}$ as a function of the LO power.

Due to environmental noise disturbances, such as the low-frequency noise of laser and relaxation oscillations, the power spectrum of the measured signal often exhibits pronounced noise in the low-frequency region, where quantum noise is overshadowed. We select a signal window centered at 825 MHz with a 1.4 GHz bandwidth as the true quantum entropy source for QRNG. Figure 5a shows the power spectrum within the effective bandwidth of the detectors in the dual-quadrature heterodyne detection. To verify the balance of the optical system, we measure the shot noise as a function of the LO power, as shown in Figure 5b. The first point in the figure represents electronic noise, and the linear behavior of the dual-quadrature quantum noise signals over a wide range of LO powers demonstrates the reliability of the detection system. It is emphasized that the LO power remains within the linear operating region of the photodiodes.

**Figure 6:**
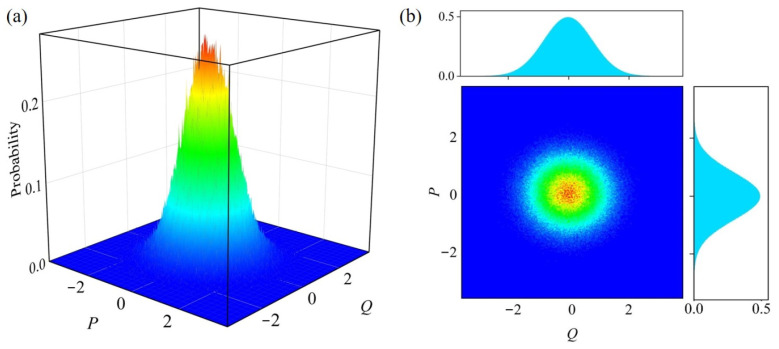
(**a**) Measured Husimi distribution of vacuum state. (**b**) The projection and marginal distributions of the measured state.

The dual-quadrature simultaneous measurement scheme not only generates quantum random numbers but also directly performs high-precision tomography of the quantum state, providing crucial evidence for the precise characterization of the entropy source. By monitoring the statistical distributions of time series and the phase space distribution of the quantum noise, the deviations from the ideal distributions can be calculated to identify potential security loopholes. We experimentally reconstruct the Husimi phase-space distribution of the vacuum state to monitor the deviations of the dual quadratures. Figure 6 shows the phase space of the vacuum state, obtained directly from the measured quadratures P and Q with the phase space resolutions of $\delta_{p}=1.477\times 10^{-4}$ and $\delta_{q}=1.472\times 10^{-4}$. Ideally, the quadrature variance of the vacuum state is 1/2. However, in the experiment, the non-quantum-noise variances of the dual-quadrature detectors are 25.4% and 25.0%, respectively, attributed to the electronic noise of the detectors and the LO fluctuations. Hence, the actual measured variances in the results are always greater than 1/2, amounting to $\sigma_{q}^{2}=0.6503$ and $\sigma_{p}^{2}=0.6663$. Moreover, we employed the NIST 800-90B entropy evaluation test to assess the entropy of the dual-quadrature raw signals. The minimum entropy values, derived from the lowest values of 10 test items, were 5.767 and 5.569 per eight bits. The corresponding maximum entropies were 7.965 and 7.947. The test results indicate that the extracted dual-quadrature entropy source exhibits high-quality randomness.

**Figure 7:**
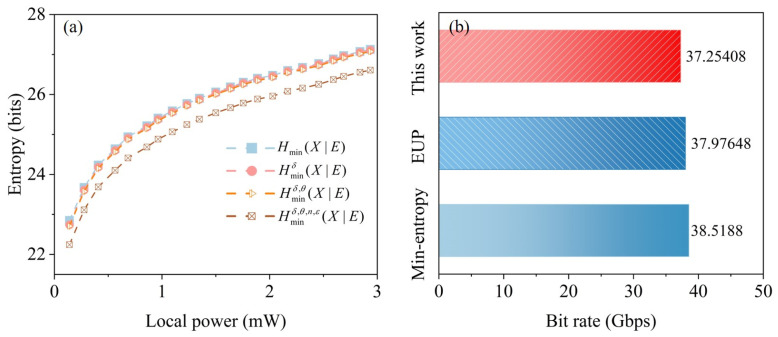
(**a**) Quantum conditional min-entropy versus LO power in the presence of imperfect factors. (**b**) Comparison of different entropy evaluation methods.

Equation (14) offers a lower bound of the secure randomness. Adjusting the LO power from 0 to 3.0 mW, we simultaneously perform $10^{6}$ independent and identically distributed measurements for the P and Q quadratures during one round of experiments, and 1/10 of the total measurements are selected for entropy evaluation. During the data acquisition process, the dual-quadrature data of heterodyne detection are selected according to the measurement, where $\theta =5^{\circ }$, $n=10^{6}$, and $\varepsilon =10^{-20}$. The maximum phase error of the optical hybrid is $5^{\circ }$. We select the maximum phase deviation and a superior security parameter to ensure a more secure and rigorous lower bound of the quantum min-entropy for the entropy evaluation. We obtain the extractable quantum conditional min-entropy as a function of the LO power, considering the impacts of the LO fluctuations, imbalanced heterodyne detection, amplitude–phase overlap, finite-size effects, and security parameter. In Figure 7a, it can be observed that the overestimated random bits are effectively removed after considering non-ideal factors. The practical entropy evaluation method used in this work is quantitatively compared with the min-entropy and the initial entropy uncertainty principle. The lower bound of quantum min-entropy we used signifies a more rigorous entropy evaluation, effectively countering the impact of non-randomness and side information. In Figure 7b, after quantization of the dual-quadrature data with a 16-bit ADC, 83.16% secure bits can be extracted, resulting in an equivalent rate of 37.25 Gbps. The effective bit depth of the ADC was measured to be 13.3056 bits. Information-theoretic Toeplitz-hash post-processing is employed in QRNG. The Toeplitz-hash matrices of 4096×3405 and 4096×3407 are used to extract quantum random numbers, and the extraction efficiencies of the two quadratures achieve 83.13% and 83.19%. We also renew the seed of the Toeplitz matrices to ensure secure and efficient post-processing [46]. Compared with other recent vacuum-based QRNG [47,48], this work focuses on the treatment of quantum side information and classical side information. Although our approach offers some essential insights into specific vulnerabilities [49], it may not fully encompass all the potential side-channel attacks. The finite bandwidth of the detectors limits the achievement of higher generation rates. By employing customized integrated detectors [47] and expanding the real-time parallel post-processing solution of our research [50], it is expected to eventually achieve high-speed QRNG at the level of 100 Gbps.

### 3.2. Quantum Conditional Min-Entropy Evaluation with Deep Learning

**Figure 8:**
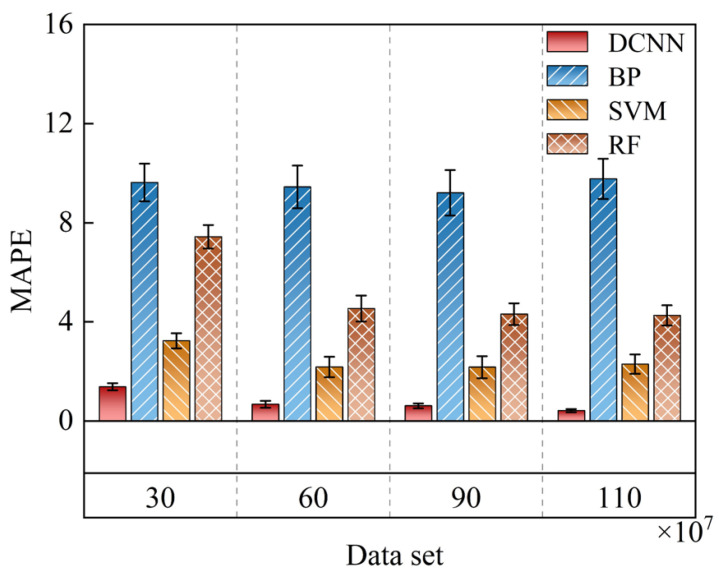
For different dataset sizes, mean absolute percentage error (MAPE) of four models.

In our work, a DCNN architecture is designed to accelerate entropy evaluation processing and enable high-speed monitoring. By continuously adjusting the weights and bias parameters, we aim to better fit the mapping relationship between the inputs and outputs. We also compare the developed DCNN model with backpropagation neural network (BP), support vector machine (SVM), and random forest (RF) models. Firstly, we investigate the impact of data volume on model accuracy. We employ four datasets of different sizes: $3\times 10^{8}$, $6\times 10^{8}$, $9\times 10^{8}$, and $11\times 10^{8}$. The test results are divided into 100 groups, each containing 134 samples. We analyze the MAPE for the four models under different data volumes, where $MAPE=\frac{100\%}{n}\sum_{i=1}^{n}\left|\frac{{\hat{y}}_{i}-y_{i}}{y_{i}}\right|$, with $\hat{y}$ representing the predicted value after the training through the DCNN and y representing the actual entropy value. In Figure 8, it is evident that, as the size of the dataset increases, the MAPE of the DCNN, SVM, and RF models gradually decreases. Moreover, the impact of dataset size on MAPE is not linear. As the random dataset size increases, the rate of error reduction slows down. Conversely, the BP model, due to its failure to capture the complex dynamical features of quantum shot noise, does not reduce its error as the dataset size increases. At a dataset size of $11\times 10^{8}$, the MAPE values for DCNN, BP, SVM, and RF are 0.4177%, 9.7689%, 2.2919%, and 4.2609%, respectively. The error bars, represented by the sample standard deviations, are 0.0654, 0.8109, 0.3906, and 0.4041, respectively. Compared to the other models, the DCNN has a lower MAPE and the smallest error range, indicating its superior learning capability in establishing the mapping between vacuum fluctuations and entropy.

**Figure 9:**
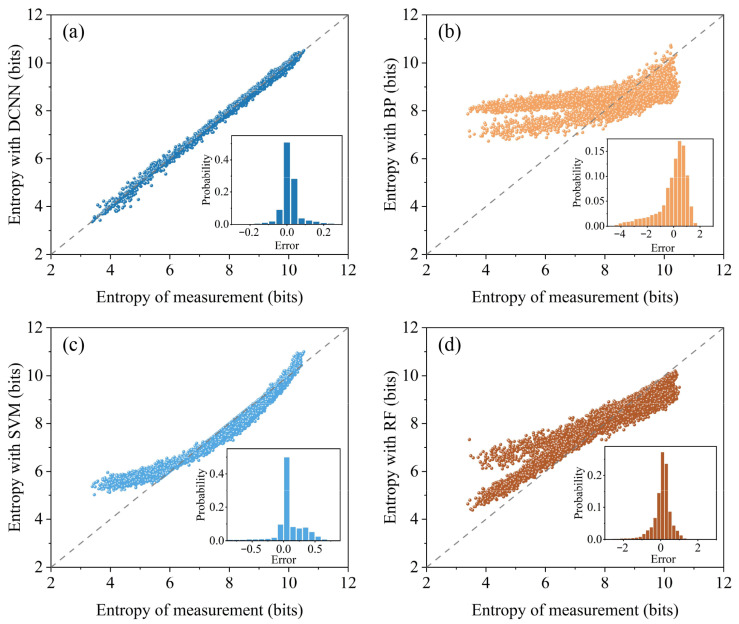
Scatter plots displaying the real entropy vs. the predicted entropy for the methods. (**a**) DCNN, (**b**) BP, (**c**) SVM, and (**d**) RF.

The scatter plots in Figure 9a–d provide visual representations of the performance of four different modeling methods. We obtain a set of predictions using 10,000 quadrature fluctuation data and achieve 13,473 entropy predictions within 68.89 s through the DCNN method. Each set comprises 10 million 16-bit quadrature amplitude and phase data, totaling 13,473 ×10M×16 bits for the training dataset. The insets illustrate the statistical distribution of the error between the model-predicted values and the actual measured values. The diagonal lines in the scatter plots represent the differences between the computed true values and the predicted values. If the difference between the accelerated results and the experimentally measured entropy is minimal, the points in the scatter plot align closely with the diagonal line, indicating higher model accuracy as the data points distribute more uniformly around the line. For the experimentally acquired quadrature fluctuations, the results obtained using the DCNN model exhibit a uniform distribution around the diagonal line across the entire coordinate range, demonstrating higher predictive accuracy. In contrast, under the same dataset training conditions, the SVM and RF methods only provide good results in certain entropy ranges, and overall their scatter plots show less alignment with the diagonal line. On the other hand, the BP method fails to capture the complexity of the dynamics of experimentally acquired quadrature fluctuations, resulting in its scatter plot also lacking alignment with the diagonal line.

**Figure 10:**
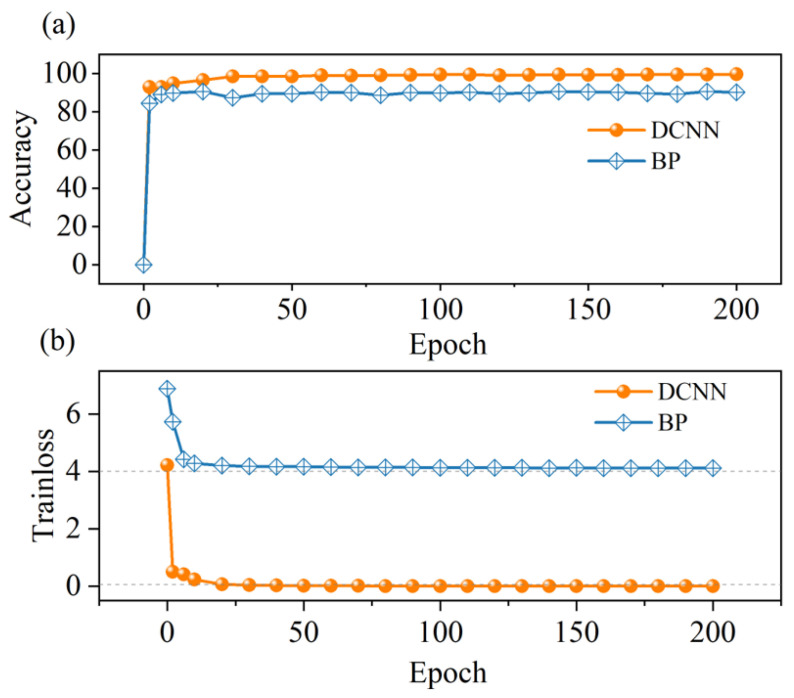
(**a**) Estimation accuracy and (**b**) training loss with epoch number for DCNN and BP deep learning models.

Meanwhile, the performance of two deep learning models in accelerating entropy assessment is compared. Figure 10a,b illustrate the test accuracy and training loss for the DCNN and BP models as functions of training epochs. The results indicate that, as the training epochs increase, both models gradually achieve maximum accuracy. The DCNN model reaches a higher accuracy of 98.58% compared to the BP model’s 90.23%. Furthermore, after 200 training epochs, the loss function value of the BP model tends to plateau at 4, indicating its failure to further reduce loss and capture the complex features of vacuum-fluctuation random sources effectively. In contrast, the DCNN model achieves a loss value of 0.05 after 50 epochs and continues to converge towards 0 with further training, further confirming its capability to better learn and represent the characteristics of vacuum-fluctuation entropy. The DCNN entropy assessment model exhibits superior performance in terms of accuracy, evaluation speed, and prediction uniformity. It achieves a faster entropy evaluation speed and has a lower MAPE. Consequently, it enables more accurate acceleration of the entropy evaluation process. Beyond clearly accelerating the entropy evaluation, the DCNN technique developed here exhibits scalable application potential. By predicting and analyzing the side information and non-ideal factors, the DCNN model, coupled with the real-time phase-space reconstruction, can rapidly identify deviations induced by imperfections or potential attacks. Furthermore, it can indirectly enhance security by dynamically adjusting parameters or applying error-correction strategies, and provide an effective way for adaptive and highly reliable QRNG.

### 3.3. Correlation and NIST Tests

**Figure 11:**
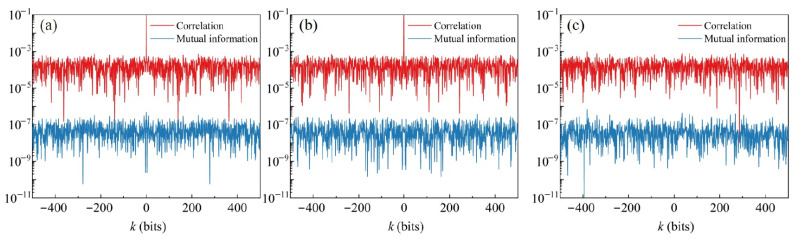
Correlation coefficient $\rho_{xy}\left[k\right]$ and mutual information $I_{xy}\left[k\right]$ of (**a**) Q quadrature, (**b**) P quadrature, and (**c**) P and Q quadratures.

To verify the independence between the two channels of the dual-quadrature heterodyne detection and the quality of the generated quantum random numbers, we analyze the correlation coefficient $\rho_{xy}$ and mutual information $I_{xy}$ between the quantum random numbers of the two channels, as shown in Figure 11. The autocorrelation and mutual information of the individual quadratures P and Q are shown in Figure 11a and Figure 11b, respectively. The cross-correlation and mutual information between the P and Q quadratures are depicted in Figure 11c. The results indicate that, as the delay *k* between the two channels increases from −500 to 500, the correlation coefficient $\rho_{xy}\left[k\right]$ and mutual information $I_{xy}\left[k\right]$ remain below the magnitudes of $10^{-3}$ and $10^{-6}$, respectively, and satisfy the condition $I_{xy}\left[k\right]≃\rho_{xy}{\left[k\right]}^{2}$. This demonstrates that there is a very low correlation between the channels. Additionally, we assess the randomness of the quantum random numbers using the NIST statistical test suite. At a significance level of α=0.01, the *p*-values for all 15 tests exceed 0.01, and the proportions are within the confidence interval of 0.99±0.00944, indicating that the random numbers successfully pass all the NIST tests. The detailed test results are presented in Table 1.

## 4. Conclusions

In summary, we introduce a scheme and protocol aimed at enhancing the efficiency and security of QRNG through deep learning-based heterodyne detection to simultaneously measure the amplitude and phase quadrature fluctuations of vacuum shot noise. Despite the imperfections in the practical implementations, our comprehensive analysis elucidates the impact of these imperfections on system security. We demonstrate that factors such as imbalanced heterodyne detection, amplitude–phase overlap, finite-size effects, and security parameters can collectively result in the overestimation of a system’s intrinsic randomness. To enhance the practical security of QRNG, we further propose the quantum conditional min-entropy lower bound. With a high-security parameter of $10^{-20}$, following a 16-bit ADC and a 1.4 GHz bandwidth extraction, QRNG achieves a quantum random number extraction rate of 83.16%, corresponding to a real-time speed of 37.25 Gbps. The randomness correlation coefficient derived from dual-quadrature measurements is below $10^{-3}$, and the result successfully passes all the NIST test items. Furthermore, to evaluate entropy rapidly and accurately, we develop a DCNN. In contrast to alternative models, our approach demonstrates superior accuracy in assessing the entropy of quantum quadrature fluctuations, with an MAPE of 0.004. It also accelerates the speed of entropy assessment by two orders of magnitude compared to traditional non-deep learning techniques, achieving 13,473 sets of quadrature data entropy evaluations within 68.89 s. This research boosts the practical applicability of QRNG and facilitates rapid and precise entropy evaluation.

## Figures and Tables

**Table 1:** NIST test results.

Test’s Name	*p*-Value	Proportion	Result
Frequency	0.16261	0.9876	Passed
Block-frequency	0.21792	0.9924	Passed
Cumulative-sums	0.01046	0.9843	Passed
Runs	0.90963	0.9821	Passed
Longest-run	0.42735	0.9894	Passed
Rank	0.04705	0.9885	Passed
FFT	0.1418	0.9830	Passed
Non-Overlapping-Templates	0.0115	0.9820	Passed
Overlapping-templates	0.22104	0.9923	Passed
Universal	0.8868	0.9998	Passed
Approximate-Entropy	0.63712	0.9856	Passed
Random-excursions	0.29161	0.9921	Passed
Random-excursions-variant	0.03263	0.9877	Passed
Serial	0.20285	0.9876	Passed
Linear-complexity	0.48334	0.9838	Passed

## Data Availability

The data that support the findings of this study are available upon reasonable request from the authors.

## Abbreviations

The following abbreviations are used in this manuscript:

QRNG	Quantum Random Number Generation
DCNN	Deep Convolutional Neural Network
ADC	Analog-to-Digital Converter
MAPE	Mean Absolute Percentage Error
LO	Local Oscillator
